# Mechanism of Rapid Curing Pile Formation on Shoal Foundation and Its Bearing Characteristic

**DOI:** 10.3390/ma17102416

**Published:** 2024-05-17

**Authors:** Wei Li, Feng Liu, Yizhong Tan, Mengjun Chen, Yi Cai, Jiayu Qian

**Affiliations:** 1 State Key Laboratory of Intelligent Construction and Healthy Operation and Maintenance of Deep Underground Engineering, China University of Mining and Technology, Xuzhou 221116, China; tbh259@cumt.edu.cn (W.L.); ly15938770068@163.com (F.L.); ts23030121a31@cumt.edu.cn (J.Q.); 2 Anhui Provincial Intelligent Underground Detection and Geoenvironmental Engineering Research Center, Anhui Jianzhu University, Hefei 230601, China; 3 Yunlong Lake Laboratory of Deep Underground Science and Engineering, Xuzhou 221008, China; 4 State Key Laboratory of Disaster Prevention and Mitigation of Explosion & Impact, Army Engineering University of PLA, Nanjing 210007, China; tanyizhong@163.com; 5 College of Future Technology, Shandong University, Jinan 250012, China

**Keywords:** rapid curing, bearing characteristic, microscopic characteristic test, on-site testing

## Abstract

This study explores the application effect of the new non-isocyanate polyurethane curing agent on the rapid curing mechanism and bearing characteristics of piles in beach foundations. Through laboratory tests and field tests, the effects of the curing agent on the physical and mechanical properties of sand were systematically analyzed, including compressive strength, shear strength, and elastic modulus, and the effects of water content and cement–sand mass ratio on the properties of sand after curing were investigated. The results show that introducing a curing agent significantly improves the mechanical properties of sand, and the cohesion and internal friction angle increase exponentially with the sand mass ratio. In addition, the increase in water content leads to a decrease in the strength of solidified sand, and the microstructure analysis reveals the change in the bonding effect between the solidified gel and the sand particles. The field static load tests of single piles and pile groups verify the effectiveness of the rapid solidification pile in beach foundations and reveal the significant influence of pile length and pile diameter on the bearing capacity. This study provides a theoretical basis and technical support for the rapid solidification and reinforcement of tidal flat foundations and provides important guidance for related engineering applications.

## 1. Introduction

Natural disasters like earthquakes, floods, and mudslides seriously threaten human society. Rapid response and effective emergency measures are essential to save lives and reduce property losses in the face of these disasters. The stability of foundations is the key factor to ensure the safety of buildings [1,2,3]. Especially when disasters occur, its importance is even more prominent. Traditional foundation reinforcement methods often take a long time, and it is challenging to meet the need for rapid reinforcement in emergencies. Therefore, developing and applying a new fast-curing agent will help to realize the rapid reinforcement of foundations [4,5,6,7,8,9]. This has become an urgent need in the engineering field.

A curing agent is a combination of chemical substances [10,11,12,13], including a hardener and liquid mixture, which is widely used to improve the engineering properties of soil [14,15,16,17,18,19,20,21,22,23,24], such as improving stability and shear strength and reducing settlement and deformation. Compared with the traditional foundation reinforcement method, the curing agent has the characteristics of rapid reaction, which can complete the curing in hours to days, significantly reducing construction time and costs [25,26,27,28]. In addition, the environmental friendliness and sustainability of curing agents are also important reasons for their popularity [29,30,31,32]. Curing agent treatment reduces the dependence on natural resources and environmental pollution and CO_2_ emissions, which aligns with sustainable development.

The application of curing agents in environmental emergency treatment cannot be ignored [33,34,35]. In oil pollution accidents, curing agents can effectively control and reduce the diffusion of oil pollutants and protect the ecosystem from damage [31]. Inland pollution accidents and curing agents can quickly immobilize harmful substances and reduce their impact on the environment and human health. These applications demonstrate the vital value of curing agents in emergencies. Seyed Javad Ramezani et al. [36] used the soil stability of glass powder and silicon powder in bagasse ash-based geopolymer and used a large amount of SCBA and GP to develop a new environmentally friendly geological curing agent. Studies have shown that curing agents can effectively improve soil strength. Ammar Abdullah Saad Al-Dossary et al. [37] used the CCR and LABSA mixture as a UGB stabilizer in pavement engineering applications. Zhu Jianfeng et al. [38] studied the macro–micro investigation of using a ternary mixture of steel slag, fly ash, and calcium carbide residue to stabilize sludge as a roadbed filler. Studies have shown that this mixture can effectively improve the stability and mechanical properties of the filler. This study is of great significance to improve the sustainability of subgrade engineering. Lian Richeng et al. [39] studied the use of Cu (II) and Co (II) complex-modified ammonium polyphosphate as a co-curing agent to improve the fire safety and mechanical properties of epoxy-based architectural coatings. Xue Chen et al. [40] used the modified curing agent to treat the geological structure of loess. The results show that the modified curing material after adding CIM can effectively improve the engineering geological properties of loess foundations. Although the curing agent has shown great potential in foundation reinforcement and environmental emergency treatment [41,42,43], there is still a lack of research on its rapid solidification mechanism and bearing characteristics in tidal flat foundations. Traditional sand solidification materials have many limitations, such as poor permeability and low early strength, which make it challenging to meet the needs of rapid reinforcement [44]. This study used a new curing agent developed by the South China University of Technology to explore its application effect in beach foundations and its influence on the bearing capacity of single piles and pile groups.

In this study, the effects of the curing agent on sand’s physical and mechanical properties were analyzed through laboratory tests, including compressive strength, shear strength, and elastic modulus. Through microscopic tests such as scanning electron microscopy, the microstructure changes of sand after curing were observed, and the bonding mechanism between the curing agent and sand particles was revealed. Then, through the static load test of a single pile and pile group, the bearing capacity of a fast-solidified pile was evaluated, and the influence of the slurry diffusion effect on the bearing capacity of the pile foundation was explored. Finally, this study will provide a theoretical basis and technical support for the rapid solidification and reinforcement of beach foundations and guidance for related engineering applications.

## 2. Curing Agent and Sample Preparation

**Figure 1:**
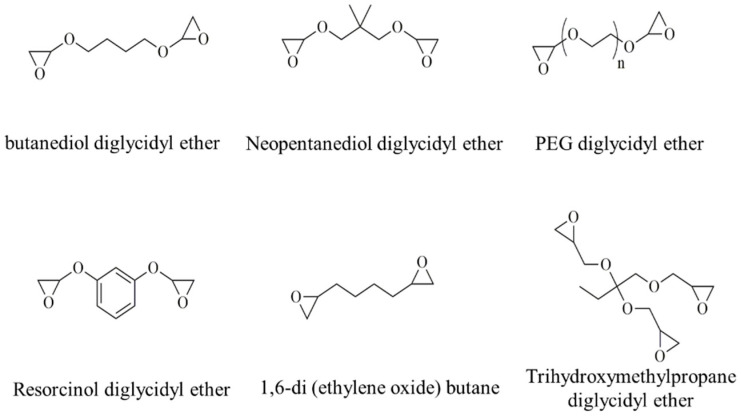
Structural formula of low-viscosity, small-molecule raw material containing epoxy group.

As shown in Figure 1, firstly, a series of low-viscosity, small-molecule compounds containing epoxy functional groups were screened as raw materials, and low-viscosity trimethylolpropane triglycidyl ether [12,21] was used as a raw material to prepare a ternary cyclic carbonate material to form a cross-linked structure to improve the mechanical strength of the final product.

**Figure 2:**
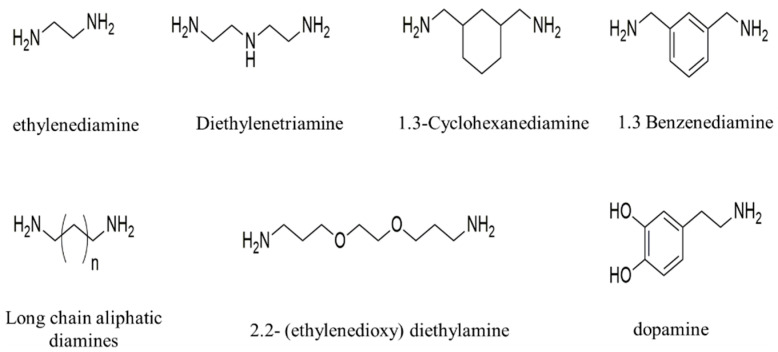
Functional curing agent.

Based on the synthesized cyclic carbonates, as shown in Figure 2, a series of functional diamines or polyamines were selected as curing agents, such as ethylenediamine, diethylenetriamine, Mannich base polyamines, dopamine, etc., to adjust the structure and composition of polyurethane materials and obtain rapid infiltration and rapid solidification polyurethane materials with excellent performance.

Subsequently, the synthesized low-viscosity cyclic carbonate was subjected to a polyaddition reaction with a small-molecule diamine to form a non-isocyanate polyurethane. During the reaction, the cyclic carbonate group reacts with the amino group causing ring-opening polymerization to form the carbamate and hydroxyl groups, enhancing polyurethane’s toughness and impact properties.

**Figure 3:**
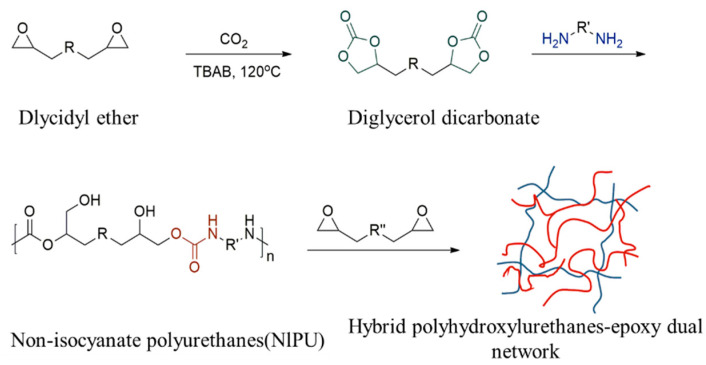
Diagram of the reaction of non-isocyanate polyurethane materials and epoxy–polyurethane materials.

Subsequently, as shown in Figure 3, by introducing a low-viscosity, small-molecule glycidyl ether material containing an epoxy functional group, an epoxy–non-isocyanate polyurethane hybrid material with a double network structure is formed in the reaction with cyclic carbonates and amines to improve the mechanical properties of the material further and enhance its permeability in the sand.

Finally, using the above process, a non-isocyanate polyurethane curing agent with low viscosity, easy infiltration, and good mechanical properties was prepared. The curing agent also has environmental friendliness, which is suitable for the engineering requirements of rapid solidification and reinforcement of sand foundation.

The sample preparation process of this study is based on the combined application of a non-isocyanate polyurethane polymer curing agent and Daqingshan beach fine sand. Firstly, the beach sand in a specific area was selected as the source of test materials and dried. Subsequently, according to the predetermined moisture content requirements, the dried sand was mixed with water for 24 h to achieve a uniform moisture distribution. In the material ratio stage, the curing agent A and B components were accurately weighed according to the established mass ratio and quickly mixed to start the curing reaction. After preliminary stirring and standing, the mixture was poured into a pre-prepared mold, and the vibration method was used to ensure that the material was fully dense. To ensure that the surface of the sample was flat and defect-free, the surface of the material after perfusion was quickly flattened and smoothed and placed in a restricted expansion device for initial maintenance. Finally, after the specified curing time, the molded sample was obtained by demolding.

Two groups of experiments were designed for this study to systematically study the influence of water content and mortar mass ratio on the physical and mechanical properties of solidified sand. The first group of experiments focused on the change in water content, while the second group focused on the change in the mortar mass ratio. In each group of tests, the samples were numbered according to different treatment conditions to be effectively distinguished in subsequent analysis and discussion. Through this detailed sample preparation process, this study aims to explore the effect of curing agents on improving sand properties under different conditions and provide a scientific basis for related engineering applications.

(1) The effect of water content on the physical and mechanical properties of solidified sand under the same mass ratio of mortar was investigated:

According to the actual moisture content of 24.26%, the moisture content of the test sand was divided into five gradients: w = 0%, 5%, 10%, 15%, 20%, 24.26%, and the mass ratio of mortar was 20.56%, 33%. The total size of the sample prepared in the test is 1 diameter × height = 50 mm × 100 mm, see Table 1.

(2) Under the same water content, the influence of the mass ratio of mortar on the physical and mechanical properties of solidified sand was explored:

According to the mortar mass ratio of 5%, 10%, 15%, 20.56%, 25%, 33% (of which 20.56% and 33% correspond to the minimum void ratio and the ratio under the maximum void ratio, respectively), the moisture content was 24.26% of the real moisture content under the field condition, so the specimens were divided into six groups. A total of two sample sizes were prepared in the test: 1 diameter 50 mm, height 100 mm; 2 diameter 78.9 mm, height 100 mm; see Table 2.

## 3. Physical and Mechanical Properties and Microscopic Characteristics of Solidified Sand

### 3.1. Compressive and Shear Properties

**Figure 4:**
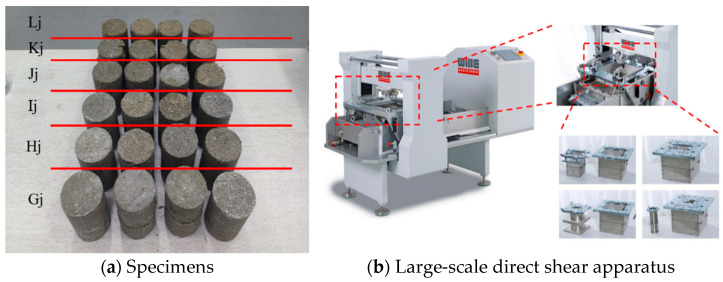
Straight shear test.

Using the ADS-500/HL-200 large-scale direct shear test system produced by WILLE Geotechnik in Rosdorf, Germany for underground structures shown in Figure 4, this study aims to evaluate the effect of curing agents on the properties of sand. The tests were carried out at different mass ratios of 5%, 10%, 15%, 20.56%, 25%, and 33%, of which 20.56% and 33% represent the minimum and maximum void ratios, respectively. The water content used in the test was 24.26%, which was based on the field measurement results. Under constant water content, the shear performance and the changing trend of samples with different mass ratios were studied to simulate the actual influence of solidified pile slurry diffusion on the mechanical properties of surrounding sand.

**Figure 5:**
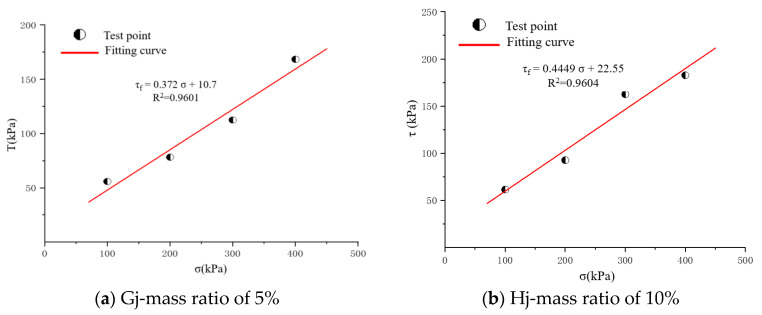

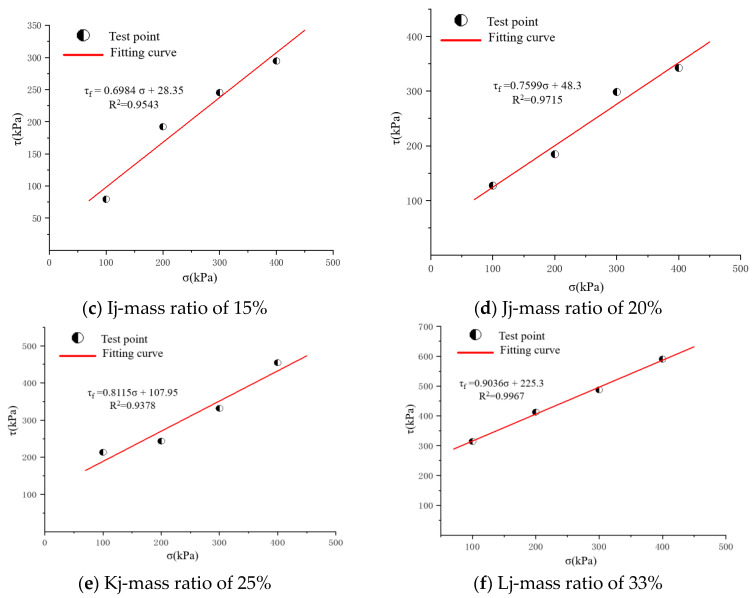
The fitting curves of shear stress (σ) and normal stress (τ).

After the test, the relationship between the shear strength and the vertical pressure of the samples with different mass ratios under the same water content was obtained, as shown in Figure 5. For detailed data, refer to Table 3.

The direct shear test results can be summarized, as shown in Table 4.

**Figure 6:**
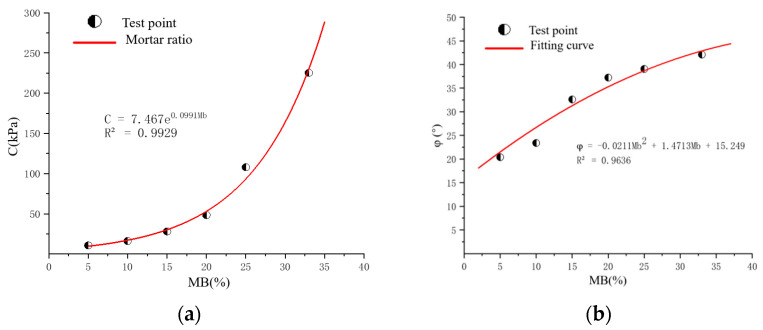
Fitting curves. (**a**) Fitting equation of the relationship between cohesion and mass ratio. (**b**) Fitting equation of the relationship between internal friction angle and mass ratio.

According to the above data, the relationship equation between cohesion and the internal friction angle and mass ratio can be obtained. The fitting results are shown in Figure 6.

As shown in Figure 6, the cohesion of the solidified sand sample has an exponential line function relationship with the mass ratio of sand to rubber, and the internal friction angle has a quadratic function relationship with the mass ratio of sand to rubber.

**Figure 7:**
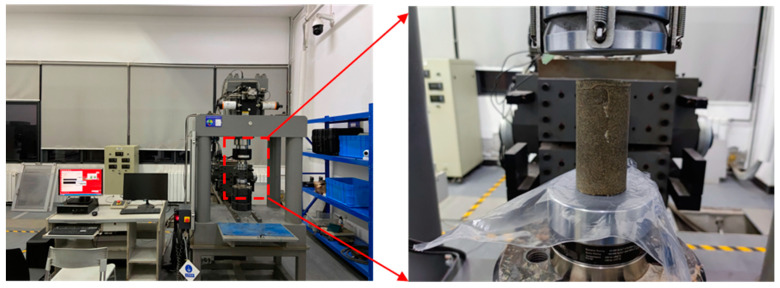
Uniaxial compression experiment.

As shown in Figure 7, the uniaxial compression test was carried out using the MTS816 electro-hydraulic servo rock mechanics test system produced by MTS System Company in the Eden Prairie, MN, USA. The sampling frequency was 10 Hz, and the pre-pressure was 0.5 kN. During the unconfined uniaxial compression test [14,26,29], Vaseline was applied to offset the possible errors in the test results due to friction forces at the interface between the faces of the specimen and the plates of the testing machine.

The influence of water content on the physical and mechanical properties of solidified sand under the same mass ratio of mortar was explored. According to the actual moisture content of 24.26%, the moisture content of the test sand was divided into five gradients: w = 0%, 5%, 10%, 15%, 20%, 24.26%, and the mass ratio of mortar was 20.56%, 33%. The sample size had a diameter of 50 mm and a height of 100 mm; the results of the uniaxial compression test are shown in Table 5.

**Figure 8:**
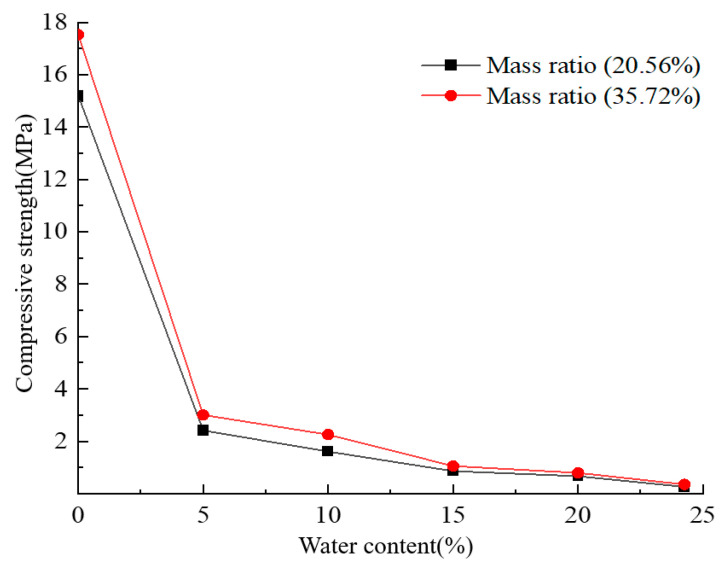
Effect of moisture content on compressive strength.

Figure 8 shows the significant effect of water content on the strength of solidified sand specimens. Under the condition of a fixed mass ratio, the strength of the specimen decreased significantly with the increase in water content. In particular, when the specimen changed from a dry sand state to a water-bearing sand state, the strength decreased sharply. For example, when the sand–binder mass ratio was 20.56%, the strength of the specimen without water content was 15.203 MPa, while the strength decreased to 2.149 MPa when the water content was only 5%. Similarly, at a sand–binder mass ratio of 33%, the strength without water content was 17.556 MPa and decreased to 3.011 MPa when the water content was 5%. In addition, when the water content increased from 5% to 24.26%, the strength of the specimens with mass ratios of 20.56% and 33% decreased from 2.419 MPa and 3.011 MPa to 0.262 MPa and 0.353 MPa, respectively. These results highlight the important influence of water content on the strength of solidified sand and suggest that the formulation of the curing agent needs to be optimized to adapt to the environment of water-bearing sand.

Under the condition of fixed water content, it was found that the increase in sand–binder mass ratio is positively correlated with the increase in specimen strength, indicating that an increase in curing agent content helps enhance the strength of the specimen. However, for two different sand mass ratios, its effect on the strength of the specimen is not significant, especially in the water-bearing sand; the change in the sand–binder mass ratio has a limited impact on the strength. The experimental data show that the specimens with higher sand–binder mass ratios always perform better with regard to strength under the same moisture content.

**Figure 9:**
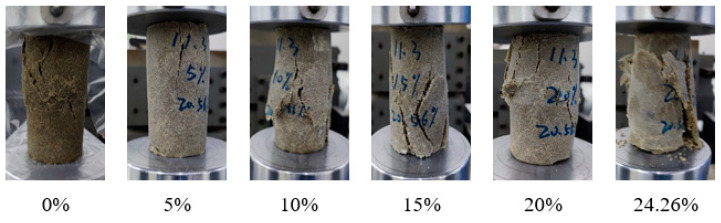
Specimen damage pattern.

Figure 9 shows the effect of different water content on the failure mode of the solidified specimen at a 20.56% mortar mass ratio. Under the condition of low water content, the non-isocyanate polyurethane curing agent can better adhere to the sand particles, thereby enhancing the strength of the specimen and forming obvious shear bands during failure, showing ‘X’-shaped or ‘Y’-shaped cracks. With the increase in water content, the gel formed by the curing agent decreases, which leads to an increase in sand particle exposure, a decrease in compressive strength, and a change in failure mode into end bulging fracture. When the water content continues to increase, the supersaturated state leads to poor adhesion of the curing agent.

With the loss of water, the compressive strength of the specimen was further weakened. Under continuous load, the internal structure of the specimen disintegrated, resulting in broken small particles accompanied by surface drainage. The failure mode of the specimen is directly related to the adhesion effect of the curing agent gel and the sand particles.

To evaluate the influence of the mass ratio of mortar on the physical and mechanical properties of solidified sand under constant water content, the specimens were divided into six groups with mortar mass ratios of 5%, 10%, 15%, 20.56%, 25%, and 33%, respectively, of which 20.56% and 33% correspond to the minimum and maximum void ratios. In the experiment, the water content of the actual working condition was 24.26%, and two specifications of the samples (diameter × height of 50 mm × 100 mm and 78.9 mm × 100 mm, respectively) were prepared. Each sample was uniquely numbered according to the working conditions. For detailed results, refer to Table 3.

**Figure 10:**
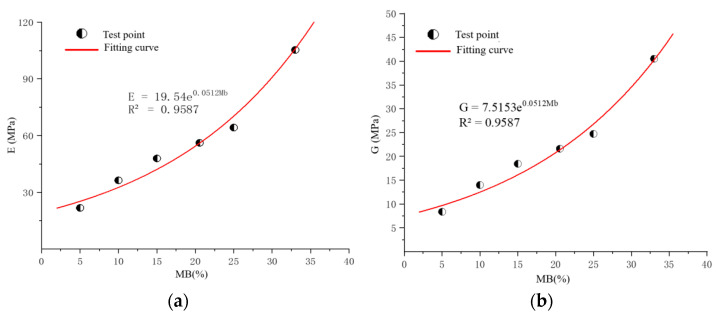
Fitting curves. (**a**) The relationship between mass ratio and elastic modulus. (**b**) Relationship between mass ratio of mortar and shear modulus.

The experimental data reveal that the elastic modulus of the sample increased with an increase in the mass ratio of the mortar, and the corresponding shear modulus could be calculated. As shown in Figure 10, an exponential growth relationship exists between elastic modulus and shear modulus and mass ratio. According to this relationship, the modulus value at any mass ratio can be predicted, or the mass ratio can be deduced from the known modulus. The results show that the increase in the concentration of the curing agent helped to improve the bonding performance of the sand, thereby enhancing its compressive and shear strength, and a corresponding mathematical model was established to describe this relationship. Detailed values are shown in Table 6.

### 3.2. Micro Characteristic Test

In this study, the microstructure of solidified sand particles was analyzed in detail by electron microscope scanning technology [27,28,29,30], with special attention given to the distribution of pores and cracks. The microstructure analysis of 12 samples was carried out under two different cement–sand mass ratios and six different water content conditions, aiming to reveal the influence of cement–sand mass ratio and water content on the microscopic characteristics of solidified sand, and further understand the formation mechanism of fast-solidified piles. The initial scanning used a magnification of 70 times to obtain the overall microstructure of the sample. Then, the key areas were observed by increasing the magnification, focusing on the microscopic morphology of holes and cracks. The test numbers for specimens with different water content and mass ratio are presented in Table 1.

**Figure 11:**
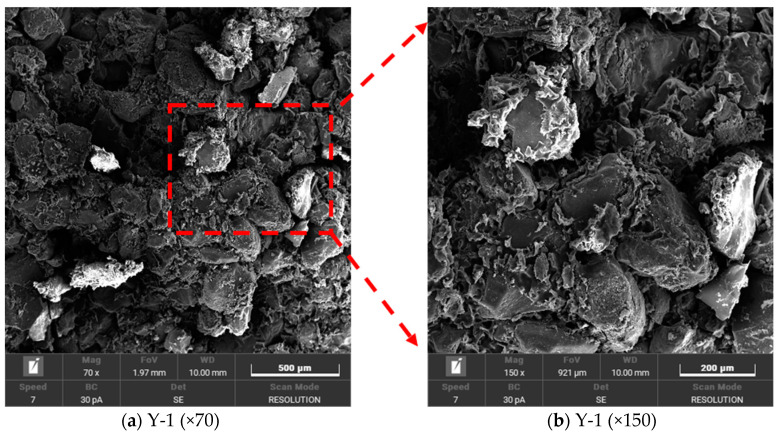
Microstructure of sandy soil after curing.

Figure 11 shows that the gel formed after the reaction of the curing agent completely or partially encapsulates the sand particles, and the voids between the particles are filled. The filling effect of this gel promotes the mutual connection between sand particles, thus changing the transition of soil particles from a free state to a bonding state and effectively improving the overall strength of the material.

**Figure 12:**
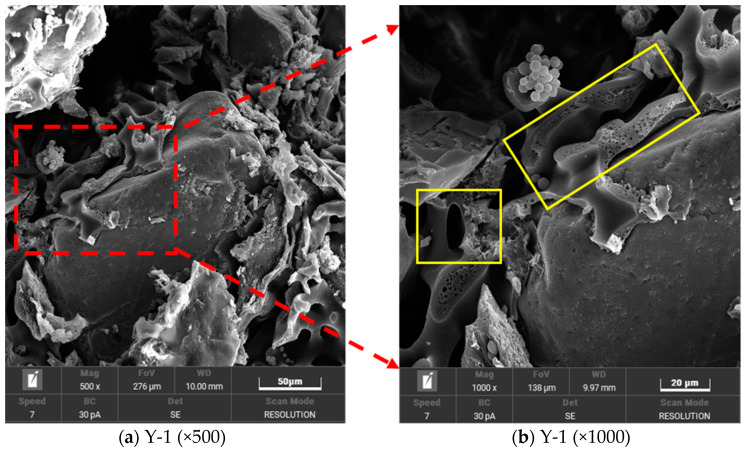
Type of microscopic pore.

It can be seen from Figure 12 that the pores existing in the solidified sand samples are divided into three types: ① intergranular pores formed by unfilled gels between sand particles; ② internal micropores formed inside the gel formed by the curing agent after curing; ③ tiny cracks formed between the gels during the adhesion process.

**Figure 13:**
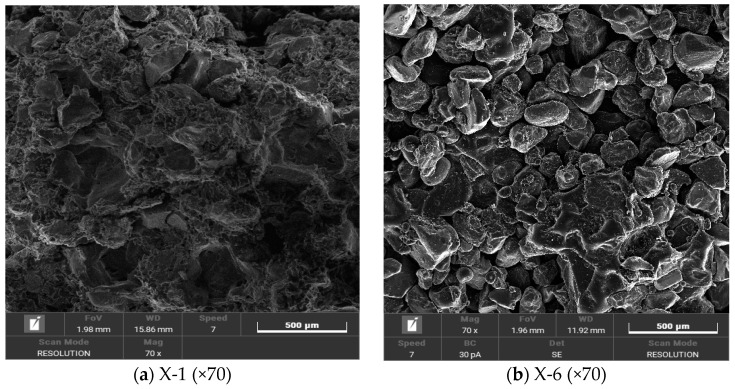

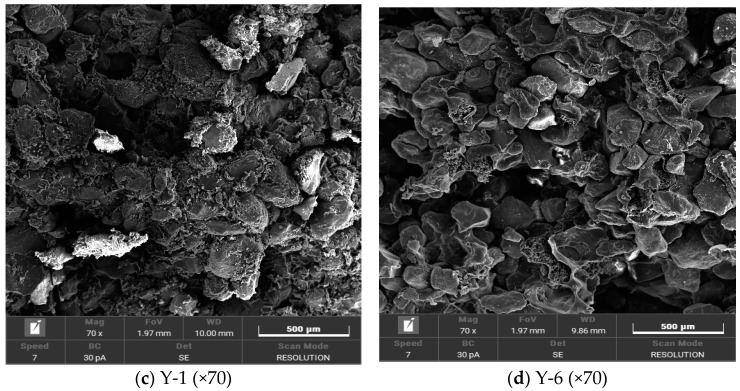
Microstructure of specimens in different states.

Figure 13 is the microstructure image of sand-solidified samples with 70 times magnification under the condition of minimum and maximum water content with two kinds of sand–binder mass ratios:

Figure 13a,b shows that under the condition of a fixed mortar mass ratio, the increase in water content leads to a decrease in the number of gels formed by the curing agent, and more and more sand particles are observed. This indicates that in the mixing process of curing agent and sand, the increase in water content may lead to a loss in curing agent with water before the formation of gel, which reduces the effective adhesion of the sand particle surface, thus reducing its ability to withstand external loads. Further, by comparing Figure 13a,c, it is found that the number of gels increases with the increase in the mass ratio of mortar, which indicates that the increase in curing agent content helps to enhance the compressive strength of the specimen. However, when the mortar mass ratio reaches 35.72%, a supersaturation phenomenon occurs in the experiment, which affects the compaction effect of the specimen. This phenomenon leads to the loss of the gel before it forms an effective bond with the sand particles, thereby weakening the overall strength of the specimen.

**Figure 14:**
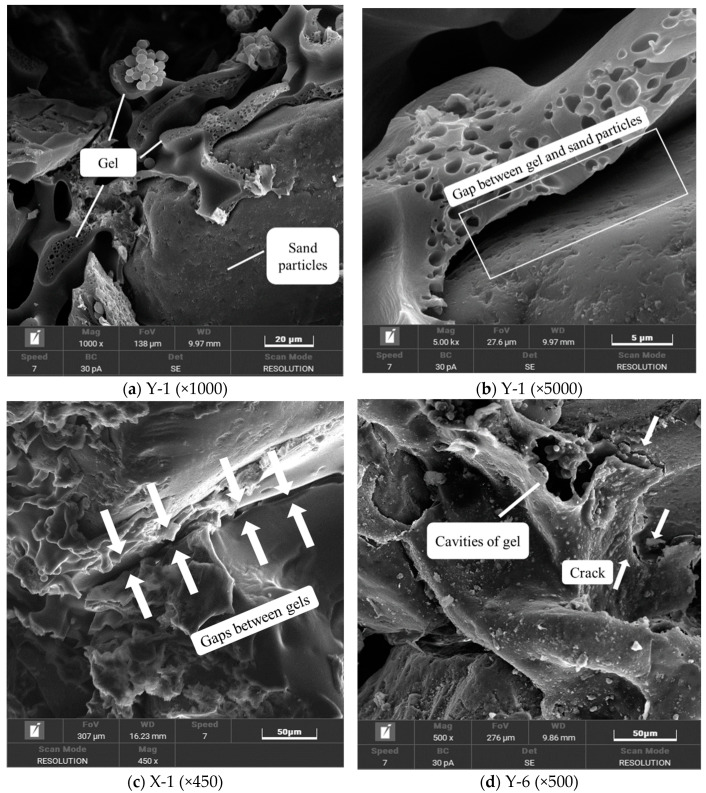
The style of specimen damage.

The microstructure diagram shown in Figure 14a reveals the fracture of the solidified gel under the action of external force, exposing its internal micro-pores and obvious fracture surfaces. Figure 14b further shows that the detachment of the gel from the surface of the sand particles leads to the exposure of some sand particles, which weakens the condensation strength and promotes the formation of the fracture surface. The formation of cracks observed in Figure 14c,d further confirms the gel rupture under external force. These observations show that the insufficient bond strength between sand particles and the gel and the insufficient strength of the gel itself to resist external forces are the main reasons for the failure of the specimen at the micro level.

**Figure 15:**
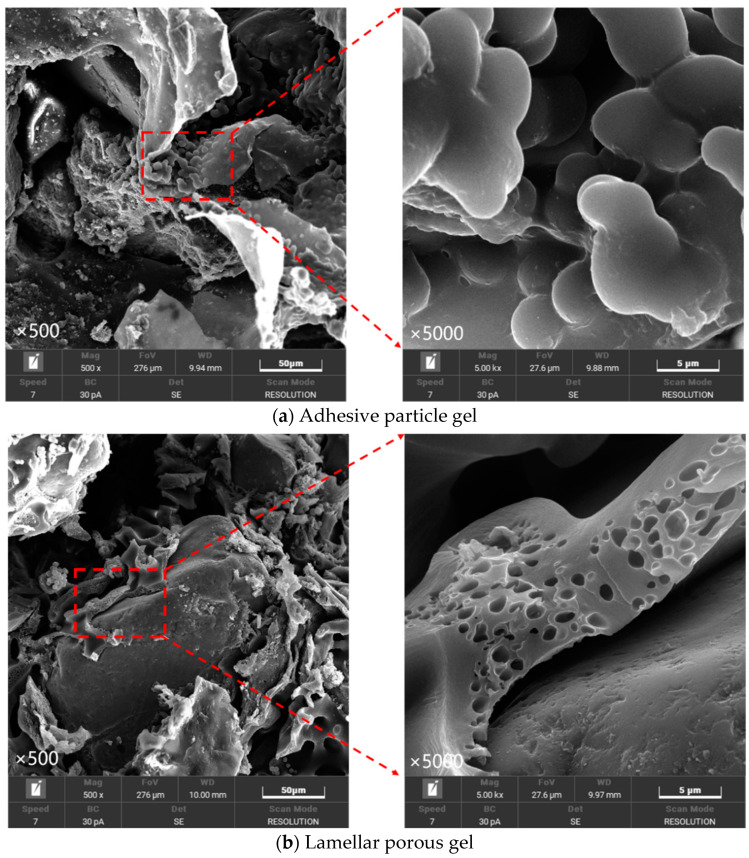
Style of solids.

As shown in Figure 15a, with the increase in water content, the gel formed by the curing agent gradually changed from a continuous dense layer to a dispersed granular structure. This transformation leads to a decrease in the bonding area between the gel particles and the dispersion of the particles, which depends on the external thin-layer cementation film to maintain the structure. Once this layer of cementation film is broken, the internal particles will lead to a sharp decline in the overall structural strength due to insufficient contact and insufficient connection with the sand particles, which will eventually lead to the destruction of the gel.

In summary, it can be seen from Figure 15b that the non-isocyanate polyurethane rapid curing agent used in this study forms a gel layer tightly wrapped with sand particles when solidifying dry, fine sand particles. It can be seen from Figure 15a that after curing in water-bearing sand, the formed gel layer structure presents a loose granular connection and is covered by a thin curing film.

## 4. Field Test of Rapid Curing Pile Foundation for a Tidal Flat Foundation

### 4.1. Single-Pile Static Load Tests

**Figure 16:**
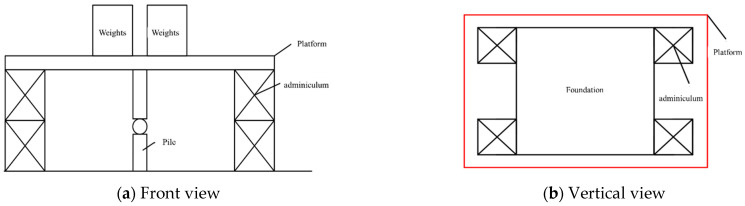
The schematic diagram of the reaction frame of the static load test pile cap.

The design strategy employs small diameters for the piles and their boreholes, with diameters set at 100 mm, 200 mm, and 300 mm. The lengths of the piles are designed to be 1 m and 0.5 m. For each mixing of the piling material, approximately 9 kg of sand is used, and the pile is cast in several stages. Utilizing a novel curing material in this experiment, the work condition design is based on a previous research framework, as detailed in Table 7. The loading approach incorporates a pile load-bearing platform and a reaction frame test arrangement, as illustrated in Figure 16.

(1) Vertical static load tests of pile P_1-1_ and pile P_1-0.5_

**Figure 17:**
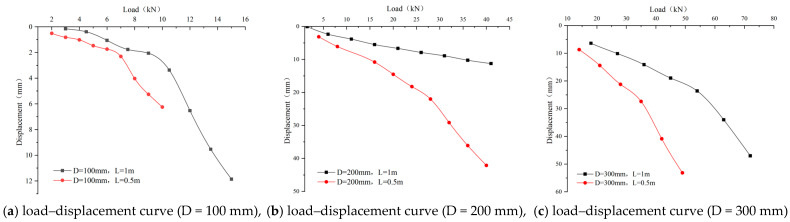
Load–displacement curve (D = 100, 200, 300 mm).

The static load test results of both pile P_1-1_ and pile P_1-0.5_ are shown in Figure 17a in the form of a load–displacement curve. The data in the figure show that there is a linear relationship between the load and settlement displacement at the initial loading stage for both test piles.

For pile P_1-1_, the curve tends to flatten when the pile top load exceeds 10.5 kN, indicating a significant increase in pile top settlement. Specifically, when the load reaches 12 kN, the settlement reaches 7.37 mm. As the load continues to increase, the curve shows a significant change, with the load reaching 13.5 kN and the settlement sharply increasing to 9.53 mm, indicating a drastic change in the pile top load–displacement curve. Therefore, it can be determined that the ultimate bearing capacity of the P_1-1_ pile is at least 10.5 kN.

However, the curve of pile P_1-0.5_ tends to flatten when the load exceeds 7 kN, indicating a significant increase in pile top settlement. Specifically, when the load reaches 8 kN, the pile top settlement is 4.03 mm. With a further increase in load, the curve shows a notable change; when the load reaches 9 kN, the settlement sharply increases to 5.26 mm, indicating a drastic change in the pile top load–displacement curve. Therefore, it can be determined that the ultimate bearing capacity of the P_1-0.5_ pile is at least 7 kN.

(2) Vertical static load tests of pile P_2-1_ and pile P_2-0.5_

Figure 17b presents the static load test results for the test piles with diameter of 0.2 m and the corresponding load–displacement curve.

Combining the above Figure 17b and load data, the pile P_2-1_ shows a relatively flat curve change after the load exceeds 31 kN, indicating a significant increase in pile top settlement. Specifically, when the load increases to 36 kN, the pile top settlement reaches 17.25 mm. With a further increase in load, a significant change in the curve trend occurs; when the load reaches 31 kN, the curve sharply declines. The pile top settlement increases to 22.25 mm when the load reaches 41 kN. Based on these observations, the ultimate bearing capacity of the pile P_2-1_ is not less than 31 kN.

Figure 17b also shows a linear relationship between the load and settlement displacement for the pile P_2-0.5_ at the initial loading stage. When the load exceeds 28 kN, the curve becomes flatter, followed by a significant increase in pile top settlement. At a load of 32 kN, the settlement is 29.14 mm. As the load increases, a noticeable change in the curve occurs; when the load reaches 36 kN, the settlement sharply increases to 36.11 mm, indicating a drastic change in the pile top load–displacement curve. Therefore, the ultimate bearing capacity of the pile P_2-0.5_ can be determined to be at least 28 kN.

(3) Vertical static load tests of pile P_3-1_ and pile P_3-0.5_

Figure 17c presents the static load test data of pile P_3-1_ and pile P_3-0.5_, as well as the corresponding load–displacement curve

The data in the figure show that there is a linear relationship between the load and settlement displacement of the pile P_3-1_ at the initial loading stage. When the load exceeds 54 kN, the curve becomes flatter, followed by a significant increase in pile top settlement. At a load of 63 kN, the settlement is 34.00 mm. As the load further increases, a noticeable change in the curve trend occurs; when the load reaches 72 kN, the settlement sharply increases to 46.99 mm, indicating a drastic change in the pile top load–displacement curve. Therefore, the ultimate bearing capacity of the pile P_3-1_ can be determined to be at least 54 kN.

The illustrated results reveal a linear relationship between the load and settlement displacement of the pile P_3-0.5_ at the initial loading stage. As the load increases, the curve gradually flattens when it exceeds 35 kN, indicating significant settlement at the pile top. At a load of 42 kN, the settlement reaches 40.86 mm. The continued increase in load causes a noticeable change in the curve trend, especially when the load reaches 49 kN; the settlement sharply increases to 53.14 mm, indicating a severe turn in the pile top load–displacement curve. Therefore, the ultimate bearing capacity of the pile P_3-0.5_ is determined to be no less than 35 kN.

The above analysis shows the bearing capacity of single piles under various conditions, as shown in Table 8. During the unconfined uniaxial compression test, Vaseline was applied at both ends of the sample to offset the possible errors in the test results due to friction forces at the interface between the faces of the specimen and the plates of the testing machine.

(1) Effect of pile length on the vertical bearing capacity of a single pile.

**Figure 18:**
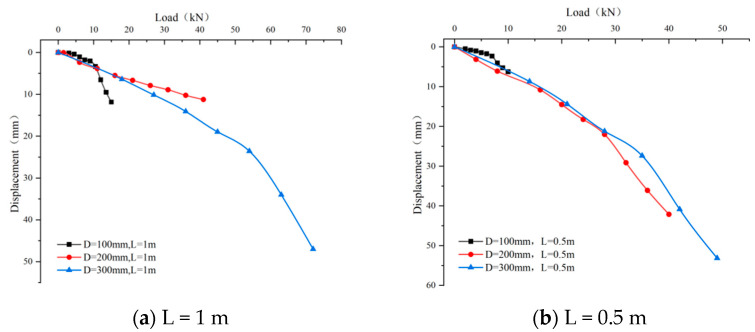
Load–displacement curve.

The load–settlement curves of single piles when the pile diameter is 100 mm, 200 mm, and 300 mm, with pile lengths of 1 m and 0.5 m, respectively, are shown in Figure 17 and Figure 18.

As is revealed in the Figure 17:

① Studies have shown that an increase in pile diameter significantly enhances the bearing capacity of pile foundations.

Assuming a pile diameter of 100 mm, when the pile top settlement is 5 mm, the bearing capacity of a pile whose length is 0.5 m reaches 9 kN, while that of a pile whose length is 1 m reaches 11 kN. Assuming a pile diameter of 200 mm, when the pile top settlement is 15 mm, the bearing capacities increase to 20 kN (L = 0.5 m) and 35 kN (L = 1 m), respectively. For a pile diameter of 300 mm, the bearing capacities increase to 40 kN and about 65 kN, respectively, when the pile top settlement is settled as 40 mm.

This phenomenon can be attributed to the increase in the contact area at the base of the pile, which increases the tip-bearing capacity of the pile. Additionally, longer piles exhibit greater load-bearing capacity as a longer pile length increases the contact area between the pile body and the surrounding soil, thereby increasing the pile-side friction resistance. In practical engineering, increasing the pile length to enhance the ultimate bearing capacity of a single pile has become a common practice due to the limitations on pile diameter.

② The inflection point of the load–displacement curve marks the ultimate bearing capacity of a single pile. The data show that, with an increase in pile length, the ultimate bearing capacity of a single pile correspondingly increases, and the settlement displacement at the pile top also increases with the increase in pile diameter. This is because pile side friction resistance exertion requires a relative displacement between the pile body and the surrounding sand soil. In the initial loading stage, the settlement of the pile foundation is small due to the low external load, resulting in the pile side friction resistance not being fully exerted.

In summary, increasing the pile length can effectively reduce the settlement of a single pile and enhance its ultimate bearing capacity, as a longer pile body increases the contact area with the surrounding soil, thereby increasing the pile side friction resistance and reducing the settlement under the same load. However, this improvement is not unlimited. Under specific geological conditions, after the pile length is increased to a certain extent, the pile top load will be fully supported by the pile side friction resistance. Once beyond this point, additional pile length will lead to excessive lateral friction resistance, causing excessive settlement, which may exceed the engineering tolerance limit for displacement. Therefore, optimizing the pile length requires balancing, improving bearing capacity, and controlling settlement.

(2) The impact of pile diameter on the vertical bearing capacity of a single pile.

Load–settlement curves for single piles with 1 m and 0.5 m lengths and diameters of 100 mm, 200 mm, and 300 mm are shown in Figure 18.

As is revealed in the figures above:

① As the pile diameter increases, the inflection point of the load–settlement curve moves towards higher loads and smaller settlements, indicating that a larger pile diameter can bring about a higher ultimate bearing capacity. For piles with a length of 1 m, under a load of 10 kN, the settlements for piles with diameters of 100 mm, 200 mm, and 300 mm are approximately 3 mm,11 mm, and 25 mm, respectively, with corresponding ultimate bearing capacities of 15 kN, 41 kN, and 72 kN.

For piles with a length of 0.5 m, under a load of 15 kN, the settlements for piles with diameters of 200 mm, and 300 mm are approximately 22 mm and 24 mm, with corresponding ultimate bearing capacities of 40 kN and 49 kN, respectively.

② The data in the figure show that, for a fixed pile length, an increase in pile diameter significantly enhances the ultimate bearing capacity of a single pile. This phenomenon is attributed to an increase in pile diameter, resulting in a larger contact area between the pile body and the surrounding soil, thereby increasing the pile side friction and pile end resistance and reducing the settlement at the pile top. Specifically, for a pile length of 1 m, the piles with diameters of 100 mm, 200 mm, and 300 mm have pile–soil contact areas of 78.5 cm^2^, 314 cm^2^, and 706.5 cm^2^, respectively. Under the same external load, a pile with a smaller diameter (D = 100 mm) experiences a greater vertical displacement than that of one with a larger diameter (D = 200 mm).

Expanding the pile diameter can enhance the maximum bearing capacity of a single pile and reduce its vertical displacement. This is mainly because the contact area between the pile tip and the soil below increases, thereby increasing the resistance at the pile tip. With the contact area between the pile body and the surrounding soil also expanding as the pile diameter increases, the lateral friction resistance increases. However, an increase in pile diameter also leads to higher costs.

### 4.2. Group Pile Static Load Tests

**Figure 19:**
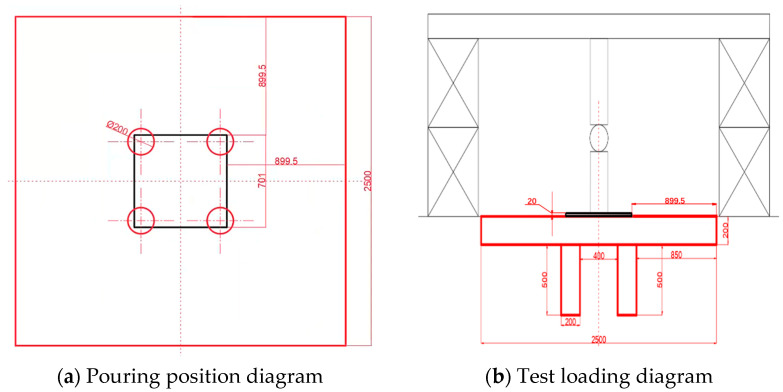
Group pile casting design drawing.

The group pile static load test selected two scenarios for comparative analysis. One scenario used a group of piles with a 200 mm thick cap at the bottom. To eliminate boundary effects, a square cap with a side length of 2.5 m was chosen, and the bearing plate used was a square with an area of 0.5 m^2^ and a thickness of 20 mm. The other scenario involved piles without a bottom solidification, using only the upper cap for the experiment. Specific conditions can be found in Table 9 and Figure 19.

(1) Analysis of test results for pile S1

**Figure 20:**
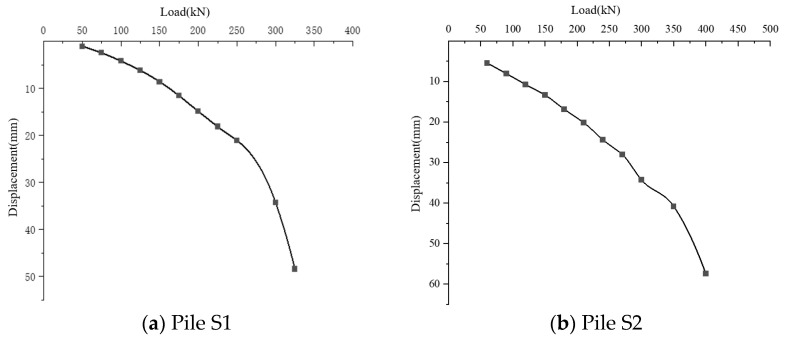
Load displacement curve.

After the experiment measurements were concluded, the pile S1 cap flat plate load results were obtained, and the load–displacement curve is illustrated in Figure 20a.

The on-site load–displacement curve shows that the vertical displacement increments at the pile top are small and uniform before a load level of 250 kN, making the curve gentle. Beyond this load, the increment in pile top displacement significantly increases, and the curve steeply declines. When the load increases to 300 kN, the displacement sharply rises, but there are no changes in the cap. As the load increases to 325 kN, the displacement further increases to 48.41 mm, and the cap plate begins to crack and sink. The slope of the curve after the inflection point is significantly steeper than before, indicating that the ultimate bearing capacity is 250 kN.

(2) Analysis of test results for pile S2

S2 group pile plate load test results with load–displacement curves are shown in Figure 20b.

The on-site load–displacement curve analysis indicates that the vertical displacement growth at the pile top is slow and uniform before the load reaches 350 kN, presenting a smooth trend whose displacement reaches 40.81 mm.

However, when the load exceeds 350 kN, the increment in pile top displacement significantly increases, and the curve steeply declines. Further increasing the load to 400 kN causes the displacement to rise to 57.44 mm sharply. The slope of the curve after the inflection point is significantly steeper than before, indicating that the ultimate bearing capacity is located at the inflection point where the load is 350 kN.

## 5. Conclusions

This study conducted a comprehensive investigation through a series of laboratory and field experiments to examine the impact of curing agents on the mechanical properties of sandy beach soils and elucidate the pile formation and bearing mechanisms of rapid-setting piles. The primary findings are summarized as follows:

(1) Adding a curing agent significantly improved the shear and compressive strength of the sandy soil. The experimental results indicate that with an increase in the mass ratio of glue to sand, both the cohesion and the angle of internal friction of the sandy soil samples exhibited a clear increasing trend, and exponential and quadratic functions can describe this growth relationship.
(2) The moisture content significantly impacts the mechanical properties of the cured sandy soil. Under the same mass ratio of glue to sand, an increase in moisture content leads to a decrease in the strength of the sandy soil samples. Moreover, the morphology and distribution of the gel formed by the curing agent after curing vary at different moisture contents, thereby affecting the microstructure and mechanical properties of the sandy soil.
(3) Field tests confirmed the viability and efficacy of rapid-curing piles in beach foundations. The ultimate load-bearing capacity of piles under various operational conditions was determined through static load tests on individual piles and plate load tests on pile groups. The impact of pile length and diameter on load-bearing capacity was thoroughly analyzed. Findings reveal that enhancing both the length and diameter of piles significantly boosts the ultimate bearing capacity of individual piles. However, this improvement is accompanied by increased settlement displacement at the pile head.
(4) The load-bearing mechanism of rapid-curing piles primarily relies on the diffusion and curing action of the curing agent slurry in the sandy soil around the pile. This process not only enhances the strength of the pile itself but also creates a certain range of influence around the pile, collectively bearing the upper load, thereby improving the overall load-bearing capacity of the pile foundation.

## Figures and Tables

**Table 1:** Specimen test number with different water ratio.

Mass Ratio	Water Content					
	0%	5%	10%	15%	20%	24.26%
20.56%	X-1	X-2	X-3	X-4	X-5	X-6
33%	Y-1	Y-2	Y-3	Y-4	Y-5	Y-6

**Table 2:** Specimen test number with different mortar ratio.

Specimen Size	Mortar Ratio					
	5%	10%	15%	20.56%	25%	33%
50 mm × 100 mm	Ad-1	Bd-1	Cd-1	Dd-1	Ed-1	Fd-1
	Ad-2	Bd-2	Cd-2	Dd-2	Ed-2	Fd-2
	Ad-3	Bd-3	Cd-3	Dd-3	Ed-3	Fd-3
79.8 mm × 100 mm	Gj-1	Hj-1	Ij-1	Jj-1	Kj-1	Lj-1
	Gj-2	Hj-2	Ij-2	Jj-2	Kj-2	Lj-2
	Gj-3	Hj-3	Ij-3	Jj-3	Kj-3	Lj-3
	Gj-4	Hj-4	Ij-4	Jj-4	Kj-4	Lj-4

**Table 3:** Uniaxial compression test results.

MB (%)	Sample	σ_p_ (MPa)	*E* (MPa)
5%	Ad-1	0.128	22.25
	Ad-2	0.126	21.11
	Ad-3	0.119	21.88
	Average value	0.124	21.74
10%	Bd-1	0.163	36.41
	Bd-2	0.167	36.55
	Bd-3	0.164	36.11
	Average value	0.165	36.35
15%	Cd-1	0.219	47.82
	Cd-2	0.213	47.98
	Cd-3	0.209	48.03
	Average value	0.214	47.94
20.56%	Dd-1	0.256	56.23
	Dd-2	0.251	55.98
	Dd-3	0.267	56.32
	Average value	0.258	56.18
25%	Ed-1	0.312	64.36
	Ed-2	0.292	64.18
	Ed-3	0.291	64.29
	Average value	0.298	64.27
33%	Fd-1	0.354	105.39
	Fd-2	0.352	105.18
	Fd-3	0.348	105.41
	Average value	0.351	105.32

**Table 4:** Summary of direct shear test results.

Mortar Ratio (%)	Cohesion (kPa)	Internal Friction Angle (°)
5	10.70	20.40
10	16.25	23.40
15	28.10	36.10
20.56	48.30	37.23
25	107.95	39.06
33	225.30	42.10

**Table 5:** Summary of test results.

No.	Compressive Strength (MPa)	No.	Compressive Strength (MPa)
X-1	15.203	Y-1	17.556
X-2	2.419	Y-2	3.011
X-3	1.612	Y-3	2.259
X-4	0.863	Y-4	1.056
X-5	0.67	Y-5	0.798
X-6	0.262	Y-6	0.353

**Table 6:** Elastic and shear modulus with different mass ratio.

Sample Property	MB (%) 5	MB (%) 10	MB (%) 15	MB (%) 20.56	MB (%) 25	MB (%) 33
E (MPa)	21.74	36.36	47.94	56.18	64.27	105.32
G (MPa)	8.36	13.98	18.44	21.61	24.72	40.51

**Table 7:** Design of working conditions for single pile.

Project	P_1-1_	P_1-0.5_	P_2-1_	P_2-0.5_	P_3-1_	P_3-0.5_
Pile diameter/m	0.1	0.1	0.2	0.2	0.3	0.3
Pile length/m	1	0.5	1	0.5	1	0.5

**Table 8:** Vertical bearing capacity of single pile.

Project	P_1-1_	P_1-0.5_	P_2-1_	P_2-0.5_	P_3-1_	P_3-0.5_
Pile diameter (mm)	100	100	200	200	300	300
Pile length (m)	1	0.5	1	0.5	1	0.5
Pile bearing capacity (kN)	10.5	7	31	28	54	35

**Table 9:** Design of working conditions for group pile.

No.	Foundation Slab	Test Pile
S1#	Side length 2.5 m	-
S2#	Side length 2.5 m	Pile diameter 200 mm, Pile length 500 mm ×4

## Data Availability

Data are contained within the article.
